# Comprehensive somatic profiling of gastroenteropancreatic neuroendocrine neoplasms

**DOI:** 10.1530/EO-26-0044

**Published:** 2026-09-24

**Authors:** Chirayu Mohindroo, Parul Agarwal, Valerie Lee, Jin He, Robert Anders, Katherine Bever, Daniel Laheru, Ana De Jesus-Acosta

**Affiliations:** ^1^ Division of Hematology, Oncology, and Blood & Marrow Transplantation, University of Iowa, Iowa City, Iowa, USA; ^2^ Department of Oncology, Abramson Cancer Center at The University of Pennsylvania, Philadelphia, Pennsylvania, USA; ^3^ Department of Oncology, Johns Hopkins University School of Medicine, Baltimore, Maryland, USA; ^4^ Department of Surgical Oncology, Johns Hopkins University School of Medicine, Baltimore, Maryland, USA; ^5^ Department of Pathology, The Johns Hopkins University School of Medicine, Baltimore, Maryland, USA

**Keywords:** neuroendocrine neoplasms, somatic variants, precision medicine, targeted therapies

## Abstract

**Background:**

The incidence of gastroenteropancreatic neuroendocrine neoplasms (GEP-NENs) is rising, yet their biological heterogeneity and variable response to treatments remain poorly understood. Comprehensive genomic characterization may uncover somatic drivers and inform biomarker-driven therapeutic strategies.

**Methods:**

We retrospectively analyzed clinically ordered next-generation sequencing (NGS) results from tumor samples of 111 patients with confirmed GEP-NENs treated at Johns Hopkins Hospital between 2020 and 2022. Pathogenic and likely pathogenic mutations were identified using OncoKB, CHASMplus, and COSMIC databases. Mutational patterns were correlated with clinical characteristics and overall survival using univariate and multivariate analyses.

**Results:**

In this retrospective study of 111 patients with gastroenteropancreatic neuroendocrine neoplasms (GEP-NENs), somatic pathogenic or likely pathogenic mutations were identified in 79% of cases. The most frequent alterations involved *TP53* (19%), *MEN1* (17%), and chromatin remodeling genes, such as *DAXX* (9%) and *ATRX* (6%). Notably, we also identified a subset of patients (9%) with mutations typically associated with hematologic malignancies. Distinct co-mutation and mutual exclusivity patterns were observed between pancreatic and non-pancreatic NENs. Poorly differentiated or high-grade tumors correlated with mutations in *TP53, KRAS*, and *CDKN2A*. Mutations in *KRAS, DAXX/ATRX*, and hematologic malignancy-associated genes were independently associated with worse overall survival.

**Conclusions:**

This study reveals distinct somatic mutation patterns in GEP-NENs associated with tumor differentiation, grade, primary site, and survival. The identification of hematologic malignancy-associated mutations in a subset of GEP-NENs suggests possible shared molecular phenotypes with poor prognostic implications. The presence of *KRAS* mutations supports exploring pan-RAS inhibitors as potential therapies in select patients. These findings highlight the clinical utility of genomic profiling in GEP-NENs.

## Introduction

The incidence of gastroenteropancreatic neuroendocrine neoplasms (GEP-NENs) has risen sharply over the past two decades (1, 2). GEP-NENs exhibit a highly variable clinical course with heterogeneous treatment responses, underscoring the need for a deeper understanding of the molecular underpinnings of GEP-NENs. In multiple tumor types, integrating molecular, signaling, and metabolic profiles has advanced precision medicine and risk stratification – enabling biomarker-driven clinical trial design, prognostication, and treatment selection (3, 4).

Both germline and somatic genomic studies have provided important insights into the underlying mechanisms of GEP-NEN pathogenesis (5, 6). Whole-genome sequencing has identified critical alterations affecting telomere maintenance, chromatin remodeling, and the mammalian target of rapamycin (mTOR) pathway (7). Beyond well-characterized genetic predisposition syndromes, such as multiple endocrine neoplasia type 1 (MEN1), Von Hippel–Lindau (VHL) syndrome, and neurofibromatosis type 1, germline testing has also revealed mutations in DNA repair genes and Lynch syndrome-associated genes (8). From a therapeutic perspective, belzutifan, a HIF-2alpha inhibitor, is now approved for patients with germline VHL mutations and represents the only FDA-approved therapy tailored to a specific genetic alteration in GEP-NENs, although these cases constitute only 1–3% of patients (6).

Despite decreasing costs and broader accessibility of next-generation sequencing (NGS), real-world data on somatic driver mutations in GEP-NENs remain scarce due to the rarity of these tumors. Accurate identification of somatic drivers is crucial to uncovering actionable targets, ultimately improving patient outcomes. Building on these insights, we hypothesize that comprehensive NGS-based somatic profiling of GEP-NENs reveals distinct genomic landscapes that drive their divergent biological behavior, clinical characteristics, and patient outcomes.

## Methods

### Study design and patients

A manual retrospective chart review was performed on consecutive patients with a histologic diagnosis of GEP-NENs evaluated in the multidisciplinary neuroendocrine clinic at the Sidney Kimmel Comprehensive Cancer Center at Johns Hopkins who underwent clinically ordered next-generation sequencing. This study was approved by the institutional review board and was conducted in accordance with the Declaration of Helsinki.

### Pathogenicity assessment and determination of deleterious mutation status

All identified mutations were cross-referenced against established mutation databases. The pathogenicity of each variant was assessed using the OncoKB database (9, 10), the MSK-IMPACT version of CHASMplus (11), and the COSMIC database (12). Only mutations classified as pathogenic or likely pathogenic according to these resources were included in the analysis. In cases where mutational specificity data were unavailable, pathogenicity was determined based on the information received from the clinical NGS report.

### Clinical outcomes

Clinical data from 2003 to 2024 were available for analysis. Clinical and pathological characteristics were collected, including age at diagnosis, gender, primary site of disease, surgical history, tumor differentiation, functional status, metastatic disease at presentation, histologic grade, and types of systemic treatments received. Metastatic status was determined based on documentation in medical oncology notes and corroborated with pathologic staging when available, consistent with standard clinical practice. Histopathologic diagnosis, grading, and differentiation were confirmed by board-certified pathologists as part of routine clinical care at our institution, in accordance with WHO classification criteria. Overall survival (OS) was defined as the time from the date of tissue diagnosis to the date of death from any cause. Patients who were alive at the time of the last follow-up were censored at their most recent contact date. The data lock date was June 30, 2024.

### Statistical analysis

Categorical variables are summarized using frequencies and percentages, while continuous variables are described using median and range or mean and standard deviation, as appropriate. Differences between continuous variables were assessed using the Wilcoxon rank sum test for two groups and the Kruskal–Wallis test for multiple groups. Associations between categorical variables were evaluated using Fisher’s exact test. Mutual exclusivity and co-occurrence of gene mutations were also assessed with Fisher’s exact test. Event-time distributions, including overall survival, were estimated using the Kaplan–Meier method. Differences between survival curves were compared using the log-rank test. Cox proportional hazards regression models were employed to estimate hazard ratios (HRs) with 95% confidence intervals (CIs) in both univariate and multivariate analyses. All statistical tests were two-sided, with a significance threshold set at *P* ≤ 0.05. Statistical analyses were performed using R software, version 4.3.2, and GraphPad Prism.

### Ethical approval and informed consent

This study was approved by the Johns Hopkins Medicine Institutional Review Board (IRB) under protocol number NA_0080582. A waiver of informed consent was granted because this was a retrospective study. All procedures were conducted in accordance with institutional guidelines and the Declaration of Helsinki.

## Results

### Patient cohort and demographics

We identified a total of 120 patients who had ordered NGS testing between 2020 and 2022. Nine patients were excluded due to insufficient tissue for NGS analysis (Fig. 1). The final cohort included 111 patients. Table 1 summarizes demographics and baseline characteristics. The median age of the patients was 59 years, and most of the patients were male (54%) and white (76%). Majority of the patients had pancreatic primaries (64%, *n* = 71). The most prevalent histologic category was well-differentiated neuroendocrine tumor (92%), and WHO grade 2 was the most common grade (45%). About 44% of the patients were metastatic at presentation, and only 21% of the patients were found to have functional tumors. NGS was performed using multiple clinically validated platforms, as summarized in Supplementary Table 1 (see section on Supplementary materials given at the end of the article).

**Figure 1:**
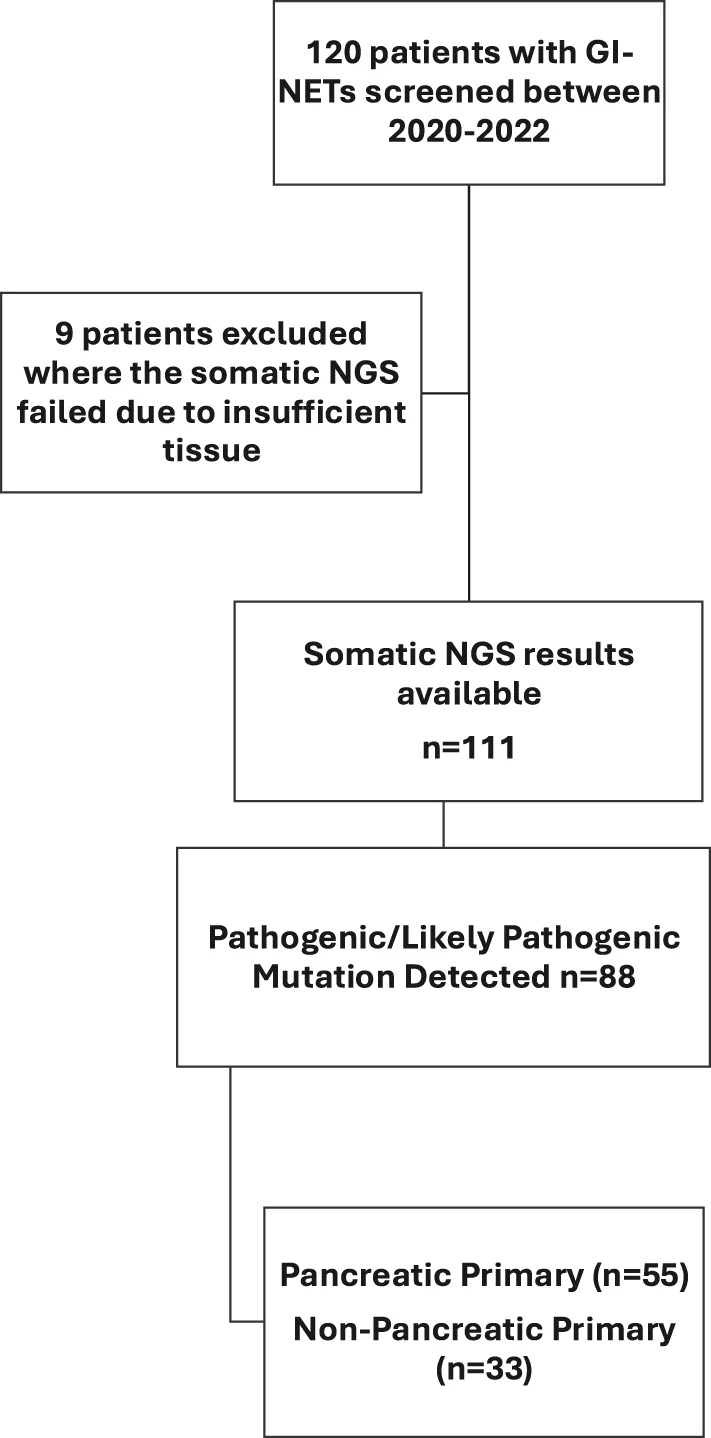
Patient cohort and somatic NGS analysis. A total of 120 patients with GEP-NENs were screened between 2020 and 2022. Nine patients were excluded because of insufficient tissue for NGS, resulting in 111 patients with available somatic NGS results. Pathogenic or likely pathogenic mutations were identified in 88 patients, including 55 with pancreatic and 33 with non-pancreatic primary tumors.

**Figure 2:**
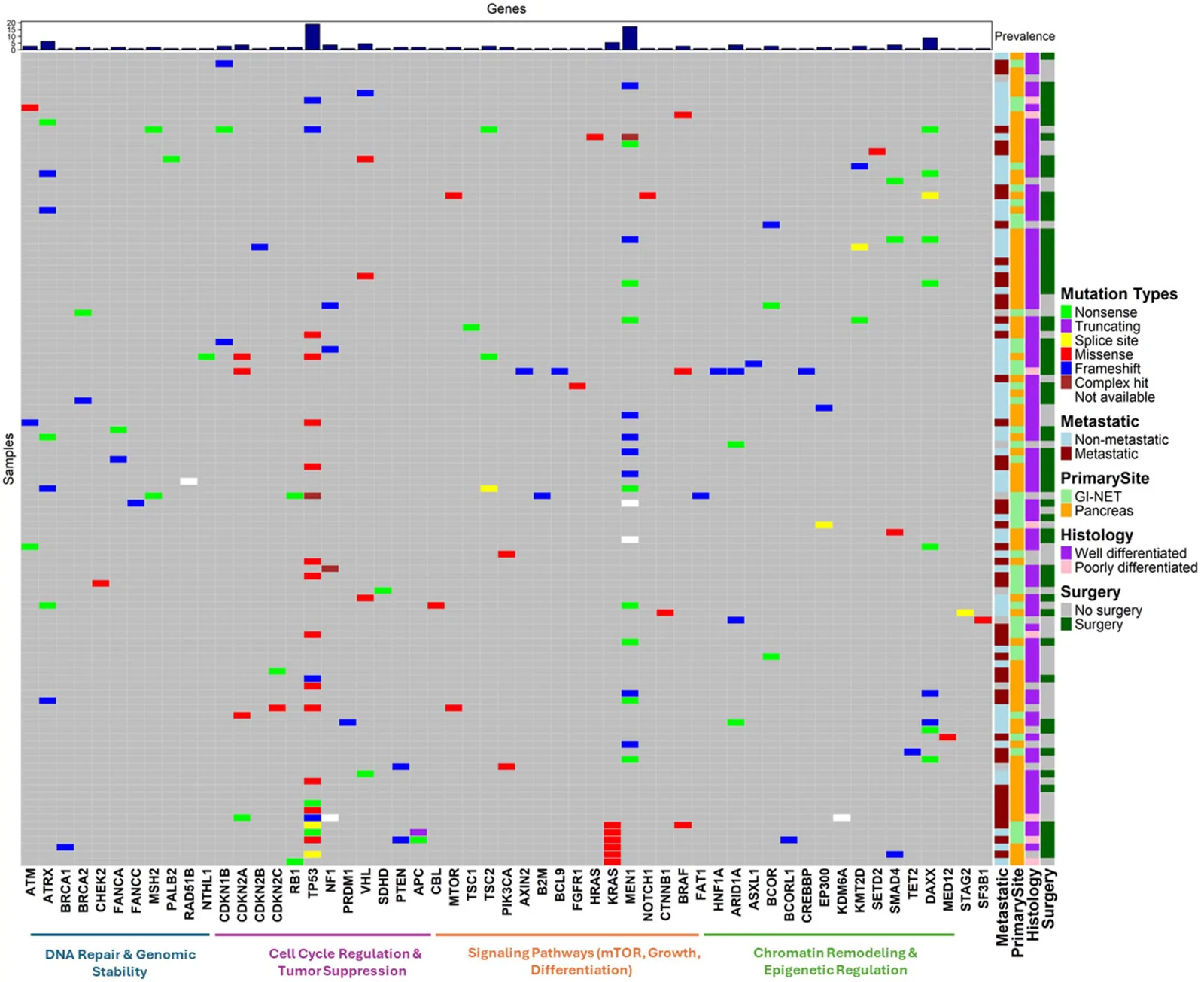
Mutational heatmap of pathogenic and likely pathogenic variants across tumors from 111 patients. Each row represents a tumor sample, and each column represents a gene. The colored tiles indicate the presence of pathogenic or likely pathogenic mutations. Gray tiles indicate unavailable data for the corresponding variable and sample, except where otherwise specified in the color legend. The heatmap illustrates the distribution and frequency of clinically relevant mutations across the cohort, alongside key clinical characteristics. Genes were grouped into broad functional categories according to their predominant biological roles described in the literature; these categories are descriptive and not mutually exclusive, as individual genes may participate in multiple biological pathways.

**Table 1:** Baseline demographic, tumor, and treatment characteristics of patients with gastroenteropancreatic neuroendocrine neoplasms.

Variable	*n* (%)
Age (*n* = 99)	
Median	58.64
Range	(21–83)
Gender (*n* = 98)	
Female	45 (46)
Male	53 (54)
Race (*n* = 78)	
White	59 (76)
Black	14 (18)
Others	5 (6)
Primary site (*n* = 111)	
Pancreas	71 (64)
Upper GI tract	7 (6)
Small intestine	16 (14)
Large intestine	7 (6)
Appendix	2 (2)
Unknown (presumed GI)	8 (8)
Histology (*n* = 95)	
Well differentiated	87 (92)
Poorly differentiated	8 (8)
Functional tumor (*n* = 96)	
Yes	20 (21)
No	76 (79)
Grade (*n* = 95)	
Grade 1	28 (30)
Grade 2	43 (45)
Grade 3	24 (25)
Metastases at presentation (*n* = 103)	
Yes	45 (44)
No	58 (56)
Surgery (*n* = 95)	
Yes	63 (66)
No	32 (34)
Systemic treatment	
Somatostatin analog (*n* = 98)	56 (57)
Everolimus (*n* = 98)	8 (8)
CAPTEM (*n* = 91)	27 (30)
PRRT (*n* = 92)	16 (17)

### Genomic landscape

#### Prevalence of mutations

Out of 111 patients, 88 (79%) had at least one pathogenic/likely pathogenic (P/LP) mutation (Fig. 2). The most prevalent mutations were *TP53* (19%), followed by *MEN1* (17%), *DAXX* (9%), *ATRX* (6%), and *KRAS* (5%). With the exception of five variants observed in four patients, detailed mutation types (such as missense, deletion, or frameshift) were available for 95% of cases.

#### Mutations based on biological landscape

Somatic alterations were grouped into four major categories based on biological function: i) DNA repair and genomic stability, ii) cell cycle regulation and tumor suppression, iii) signaling pathways, and iv) chromatin remodeling and epigenetic regulation. This was largely consistent with the current literature (7). In the DNA repair and genomic stability category, several patients harbored P/LP mutations in DNA repair genes, with potentially actionable alterations in *BRCA1* (*n* = 1), *BRCA2* (*n* = 2), *PALB2* (*n* = 1), *RAD51B* (*n* = 1), and *ATM* (*n* = 3). We also identified a notable subset (9%) of patients carrying mutations associated with hematologic malignancies, including *KMT2D* (*n* = 3), *ASXL1* (*n* = 1), *BCOR* (*n* = 2), *TET2* (*n* = 1), *BCL9* (*n* = 1), *KDM6A* (*n* = 1), *BCORL1* (*n* = 1), and *CBL* (*n* = 1) (13, 14, 15). Cellular component enrichment analysis (16) (using ShinyGO 0.82) of pathogenic mutations revealed significant involvement of nuclear and chromatin-associated complexes in both pancreatic (Fig. 3A) and non-pancreatic NENs (Fig. 3B). In pancreatic primaries, key enriched components included the TSC1–TSC2 complex, MLL3/4 complex, and DNA repair complexes, while non-pancreatic primaries showed enrichment in the beta-catenin destruction complex, Fanconi anemia nuclear complex, and DNA repair complexes. Both tumor types shared dysregulation of chromatin remodeling and DNA repair pathways, highlighting the central role of epigenetic regulation and genome maintenance. However, pancreatic primaries demonstrated stronger involvement of epigenetic modifiers, whereas non-pancreatic primaries exhibited additional alterations in Wnt signaling components.

**Figure 3:**
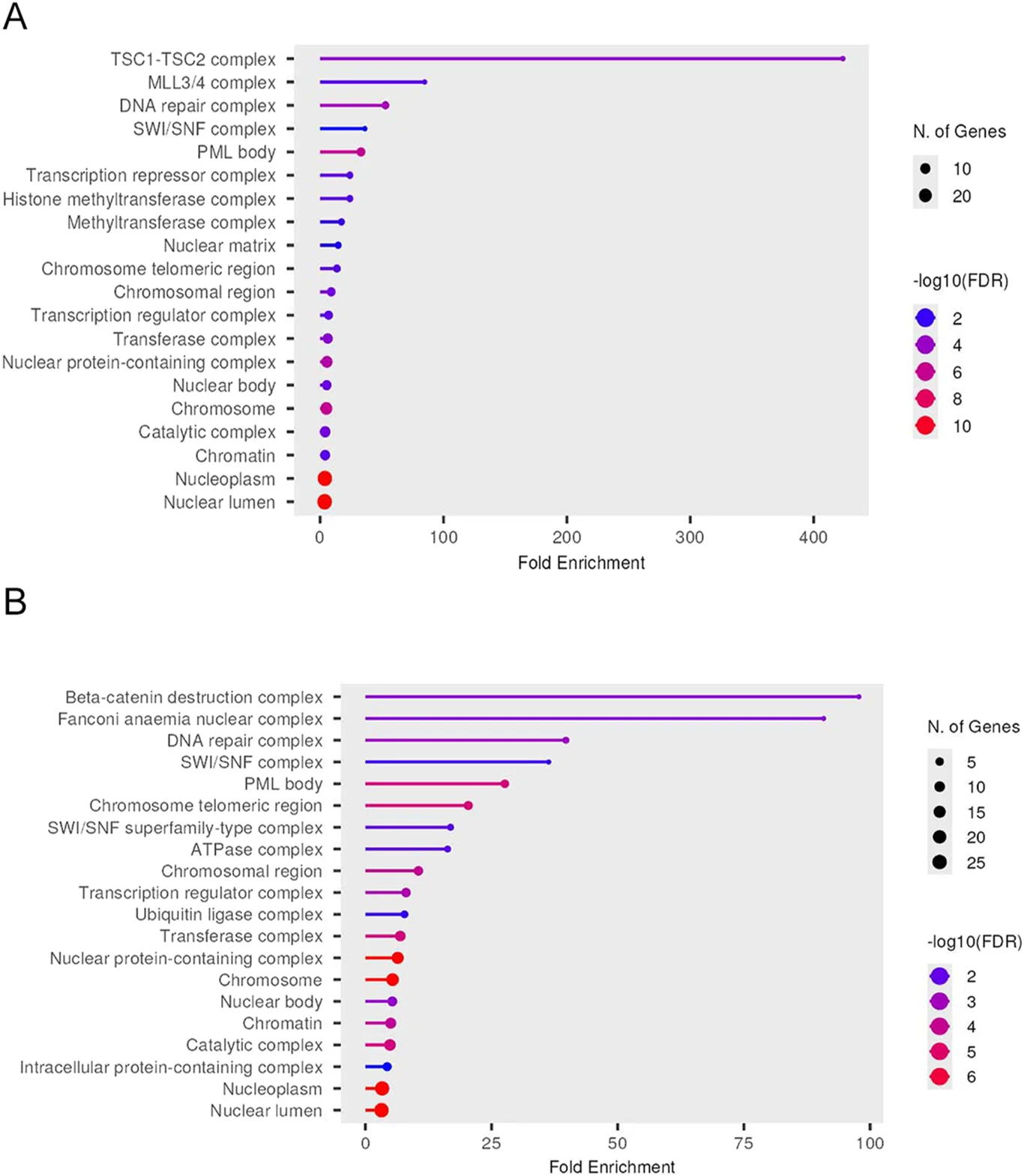
(A and B) Cellular component enrichment analysis of pathogenic mutations in pancreatic primaries (A) and non-pancreatic primaries (B). Using ShinyGO (v0.82), enrichment analysis revealed significant involvement of nuclear and chromatin-associated complexes in both tumor types. Pathways are sorted by fold enrichment (x-axis), with circle size representing the number of genes and color indicating –log10(FDR) (red = high; blue = low). The lollipop chart format illustrates both the magnitude and significance of enrichment.

### Gene co-occurrence and mutual exclusivity patterns in non-pancreatic primaries and pancreatic neuroendocrine neoplasms

We next calculated the odds ratio (OR), which quantifies the association between mutations in two genes, where OR > 1 indicates co-occurrence and OR < 1 indicates mutual exclusivity. In non-pancreatic NENs (Fig. 4A), multiple significant gene co-occurrences were observed. The strongest included *APC–KRAS* (*n* = 3; OR = 125, *P* = 0.0038) and *KRAS–TP53* (*n* = 2; OR = 52.1, *P* = 0.0035), indicating frequent concurrent mutations in key oncogenes and tumor suppressors. Additional notable co-occurrences, each observed in a single tumor (*n* = 1), with high odds ratios (OR = 237, *P* = 0.025) were seen among pairs such as *ATRX–CBL*, *AXIN2–BCL9, B2M–MSH2*, and *BCL9–CREBBP*, although these involved fewer mutation events, suggesting coordinated alterations across multiple pathways. The co-occurrence (*n* = 2) of *APC* and *TP53* was also significant (OR = 30.5, *P* = 0.027), reinforcing their critical roles in non-pancreatic primary pathogenesis.

**Figure 4:**
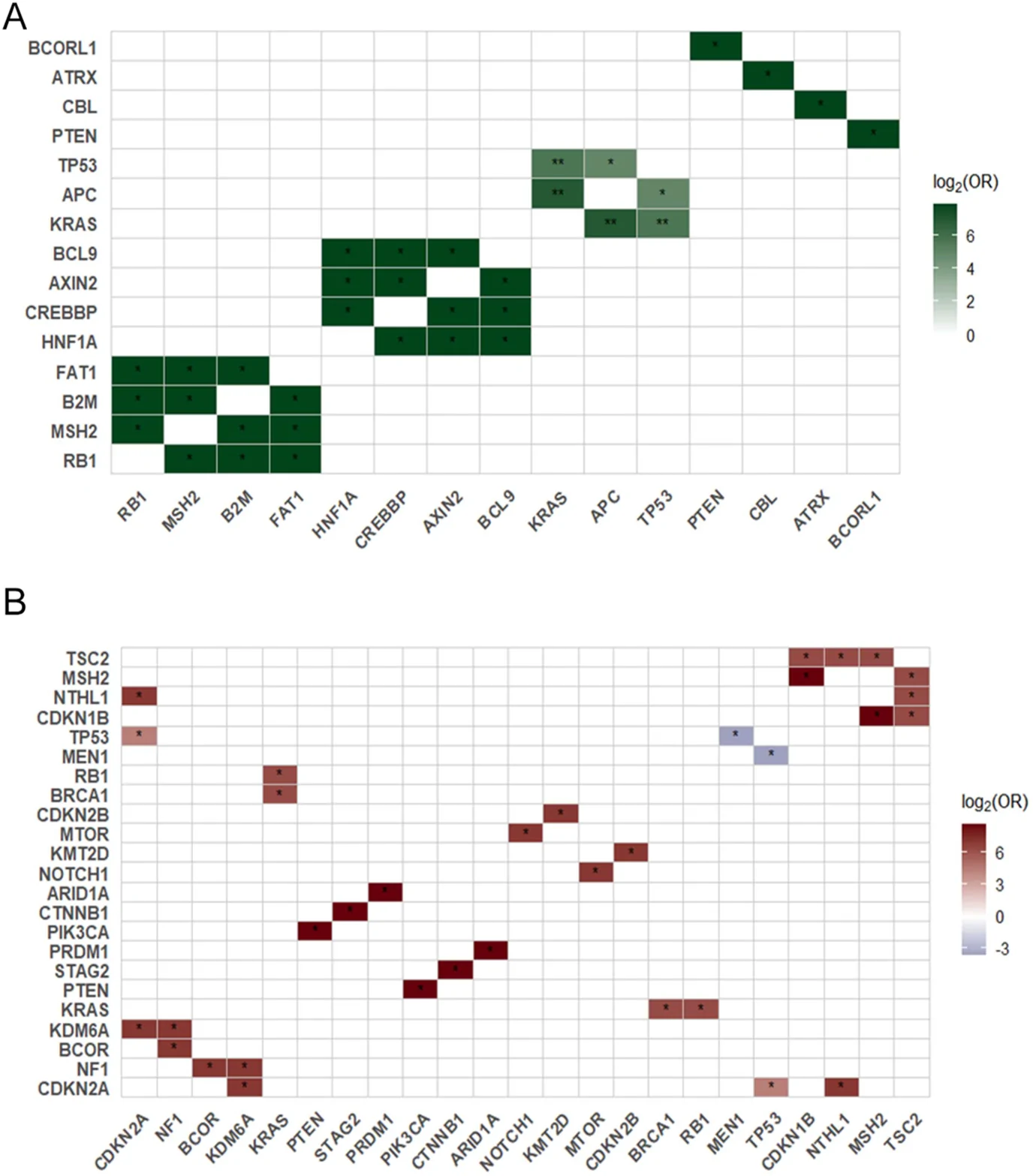
Heatmaps of significant gene pair interactions. Heatmaps show log2-transformed odds ratios (log2(OR)) for gene pairs with significant mutual exclusivity or co-occurrence. (A) Non-pancreas network: green indicates co-occurrence and light green indicates mutual exclusivity. (B) Pancreas network: red indicates co-occurrence and blue indicates mutual exclusivity. The white cells denote no significant interaction. The asterisks mark significance levels (*P* < 0.05; *P < 0.01; **P < 0.001). Genes are hierarchically clustered to reveal interaction patterns.

**Figure 5:**
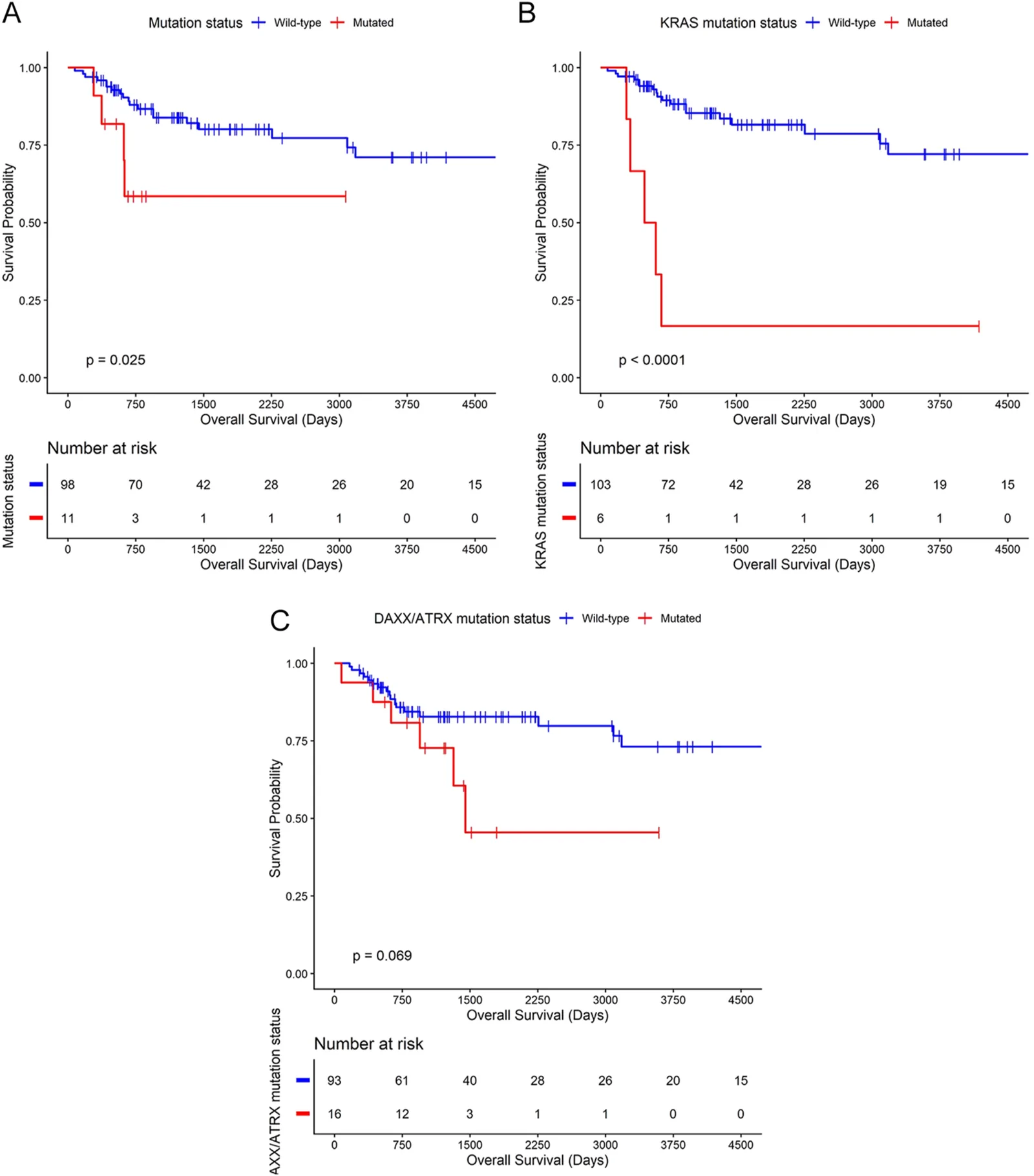
(A, B, C) Kaplan–Meier analysis of overall survival based on genomic alterations. Patients with hematologic malignancy-associated mutations (A), KRAS mutations (B), and DAXX/ATRX alterations (C) showed significantly worse overall survival. Survival differences remained significant after adjusting for clinical confounders in multivariate analysis (not shown). The curves are shown to 4,500 days for visualization; log-rank *P*-values reflect the full follow-up.

In contrast, pancreatic tumors (Fig. 4B) displayed distinct mutational patterns. Significant co-occurrences with moderate-to-high odds ratios were identified for *ARID1A–PRDM1, CDKN1B–MSH2, CTNNB1–STAG2*, and *PIK3CA–PTEN* (each *n* = 1; OR = 423, *P* = 0.014), suggesting synergistic mutations impacting chromatin remodeling, DNA repair, and signaling pathways. Notably, a significant mutual exclusivity between *MEN1* and *TP53* mutations (OR = 0.08, *P* = 0.016) suggests that these define separate molecular subgroups within pancreatic NETs. Additional co-occurrences, such as *CDKN2A–TP53* (*n* = 2; OR = 23, *P* = 0.037) and *BRCA1–KRAS* (*n* = 1; OR = 82.2, *P* = 0.042), highlight complex mutational interactions in pancreatic tumors. Given their established biological relevance in pancreatic NETs, we additionally examined MEN1–ATRX and MEN1–DAXX co-mutation patterns. MEN1–ATRX and MEN1–DAXX co-mutations were observed in three and four tumors, respectively, although neither demonstrated statistically significant co-occurrence (MEN1–ATRX: OR 3.55, *P* = 0.144; MEN1–DAXX: OR 2.49, *P* = 0.237).

Taken together, these findings reveal distinct co-mutation landscapes in non-pancreatic versus pancreatic NENs, reflecting different underlying molecular mechanisms and providing insights that may inform targeted therapeutic strategies.

### Clinical characteristics

We subsequently evaluated the association between pathogenic mutations and clinical–pathological characteristics within the cohort (Table 2). We observed the following findings:

- *TP53* mutations were significantly more likely in patients with metastatic disease (*P* = 0.004).
- Higher-grade tumors harbor distinct mutational panels. Poorly differentiated tumors were more frequently associated with mutations in *KRAS* (*P* = 0.009), *TP53* (*P* = 0.02), *BRAF* (*P* = 0.01), and *CDKN2A* (*P* = 0.03) compared with well-differentiated tumors. Similarly, patients with WHO grade 3 tumors had a higher likelihood of harboring *TP53* (*P* = 0.006), *KRAS* (*P* = 0.01), and *APC* (*P* = 0.04) mutations, compared with those with lower-grade tumors.
- When analyzing tumor primaries, pancreatic tumors were more often found to carry *DAXX* (*P* = 0.01) and *MEN1* (*P* = 0.01) mutations.
- In addition, patients with *ATRX* mutations (mean age: 67 years) were older than those with wild-type *ATRX* (mean age: 57 years) (*P* = 0.04), suggesting that *ATRX* mutations are more common in an older age group and maybe somatic variants acquired during tumor progression.

**Table 2:** Associations between clinicopathologic features and selected genomic alterations.

Gene			*P*-value
	**Metastatic at presentation (*n* = 45)**	**Localized at presentation (*n* = 58)**	
TP53	14 (31)	5 (9)	0.004
	**Poorly differentiated (*n* = 8)**	**Well differentiated (*n* = 87)**	
KRAS	3 (38)	2 (3)	0.009
BRAF	2 (25)	1 (1)	0.01
TP53	4 (50)	12 (14)	0.02
CDKN2A	2 (25)	2 (2)	0.03
	**Pancreas primary (*n* = 71)**	**Non-pancreas primary (*n* = 40)**	
DAXX	10 (14)	0 (0)	0.01
MEN1	17 (24)	2 (5)	0.01
	**Age mutated**	**Age WT**	
ATRX	67	57	0.04

	**Grade 1 (*n* = 28)**	**Grade 2 (*n* = 43)**	**Grade 3 (*n* = 24)**	***P* value**
TP53	2 (7)	5 (13)	9 (38)	0.006
KRAS	1 (4)	0 (0)	4 (17)	0.01
APC	0 (0)	0 (0)	2 (8)	0.04

Taken together, *TP53* mutations were associated with metastasis, and high-grade or poorly differentiated tumors exhibited distinct mutational profiles. Pancreatic primaries showed frequent *DAXX* and *MEN1* mutations, while *ATRX* mutations in older patients may represent somatic variants acquired during tumor progression.

### Survival analysis

To assess the prognostic impact, we analyzed mutations present in ≥5% of the cohort with available survival data using multivariate analysis. This included *KRAS* (*n* = 6), *TP53* (*n* = 21), *and MEN1* (*n* = 19). Based on the prior literature, *DAXX* and *ATRX* mutations (*n* = 16) were analyzed as a combined group (17). Using univariate analysis, *KRAS* (HR: 9.62, *P* < 0.0001) and *TP53* (HR: 2.94, *P* = 0.01) were associated with significantly lower OS. Tumors with *DAXX/ATRX* showed a trend toward worse OS although not statistically significant (HR: 2.34, *P* = 0.07). Next, we identified a set of mutations frequently observed in hematologic malignancies (*n* = 11) and homologous DNA repair (HRD) genes (*n* = 11), which were grouped together for exploratory analysis. Mutations in hematologic malignancy-associated mutations (HR: 3.31, *P* = 0.03) were significantly associated with worse OS (Table 3).

**Table 3:** Univariate and multivariate analysis of clinical and genomic factors associated with overall survival.

Variable	Univariate OS HR (95% CI)	*P* value	Multivariate OS HR (95% CI)	*P* value
Age	1.03 (0.99–1.07)	0.071	1.02 (0.98–1.071)	0.26
Histology (well vs poorly differentiated)	**10.2 (3.42–30.38)**	**<0.0001**	**9.31 (1.49–58.15)**	**0.01**
Metastatic (yes vs no)	**3.65 (1.49–8.93)**	**0.004**	2.39 (0.73–7.759)	0.14
Primary site (pancreas vs non-pancreas)	0.85 (0.36–1.96)	0.705	0.64 (0.21–1.897)	0.42
Sex (M vs F)	1.09 (0.47–2.49)	0.835		
PRRT (yes vs no)	1.62 (0.67–3.90)	0.279		
Surgery (yes vs no)	**0.27 (0.11–0.66)**	**0.004**	**0.24 (0.08–0.778)**	**0.01**
CAPTEM (yes vs no)	2.12 (0.82–5.48)	0.118		
Everolimus (yes vs no)	1.94 (0.64–5.86)	0.237		
Somatostatin (yes vs no)	0.80 (0.34–1.88)	0.616		
ATRX/DAXX (M vs WT)	**2.34 (0.91–6.01)**	**0.07**	**3.57 (1.05–12.13)**	**0.04**
KRAS (M vs WT)	**9.62 (3.71–24.96)**	**<0.0001**	**7.45 (1.64–33.81)**	**0.009**
MEN1 (M vs WT)	0.42 (0.09–1.81)	0.247		
TP53 (M vs WT)	**2.94 (1.26–6.82)**	**0.012**	1.39 (0.45–4.270)	0.55
Hematological malignancies (M vs WT)	**3.31 (1.09–10.09)**	**0.03**	**4.39 (1.09–17.69)**	**0.03**
HRD repair genes	0.89 (0.20–3.81)	0.87		

Bold indicates statistical significance in either univariable or multivariable analysis.

We evaluated potential confounding factors, including age, tumor histology, metastatic status at presentation, primary tumor site, gender, surgical resection, treatment modalities (chemotherapy, everolimus, and somatostatin analogs), and mutation status. Metastatic disease (HR: 3.65, *P* = 0.004), surgical resection (HR: 0.27, *P* = 0.004), and tumor histology (HR: 10.2, *P* < 0.0001) were identified as additional confounders (Table 3).

In multivariate analysis adjusting for these confounders, hematologic malignancy-associated mutations, *KRAS*, and the *DAXX/ATRX* group remained independently associated with worse OS. Kaplan–Meier survival curves comparing these groups are shown in Figure 5. Among patients with *KRAS* mutations (*n* = 6), three had the G12D variant, two had G12R, and one had G13D. The distribution of hematologic malignancy-associated mutations was as follows: *KMT2D* (*n* = 3), *ASXL1* (*n* = 1), *BCOR* (*n* = 2), *TET2* (*n* = 1), *BCL9* (*n* = 1), *KDM6A* (*n* = 1), *BCORL1* (*n* = 1), and *CBL* (*n* = 1).

## Discussion

In this study, we comprehensively characterized the genomic landscape of GEP-NENs using NGS from 111 patients. Our findings show distinct mutational profiles associated with tumor differentiation, site, grade, and clinical outcomes, highlighting the biological heterogeneity of these tumors and potential implications for targeted treatment and prognostication.

Consistent with previous reports, mutations in *TP53, MEN1, KRAS*, and *DAXX/ATRX* were among the most prevalent alterations detected in our cohort and demonstrated significant associations with clinical features (7, 17, 18, 19, 20). *TP53* mutations, in particular, were enriched in poorly differentiated tumors and metastatic disease (21, 22). This supports the role of *TP53* as a key tumor suppressor, whose disruption may drive aggressive tumor behavior and portend poor prognosis in GEP-NENs, especially in high-grade subtypes. Similarly, mutations in *KRAS*, *CDKN2A*, and *BRAF* were enriched in poorly differentiated and WHO grade 3 tumors, consistent with their established roles in promoting aggressive tumor biology (17, 23, 24). These findings suggest that such alterations may serve as negative prognostic biomarkers and help identify a high-risk subset of patients who could benefit from intensified surveillance or earlier therapeutic strategies.

In contrast, *MEN1* mutations were more frequently observed in well-differentiated pancreatic NENs and were associated with more favorable clinical behavior, supporting their role as early or indolent drivers within this subtype (8, 25). The mutual exclusivity of *MEN1* and *TP53* mutations in pancreatic primaries, along with the frequent co-occurrence of *CDKN2A* and *TP53*, suggests distinct evolutionary trajectories and tumorigenic pressures within this group. Notably, the older age of patients with ATRX-mutant tumors raises the possibility that these alterations may be acquired during tumor progression. Our identification of *KRAS* mutations, although less frequent compared with other studies, was notable for its strong association with poor survival outcomes (19, 20, 26). This aligns with emerging evidence implicating *KRAS* as a driver mutation in a subset of neuroendocrine carcinomas underscoring its potential as a prognostic biomarker and a therapeutic target (27). While we did not identify *KRAS G12C* mutation, case reports have shown successful treatment using sotorasib in neuroendocrine tumors (28). Additionally, we identified three patients with G12D, which is currently being targeted in other cancer types along with pan-RAS inhibitors that have shown promising responses in pancreatic adenocarcinoma (29, 30). However, these observations should be interpreted as exploratory, and the clinical applicability of KRAS-directed therapies in GEP-NENs remains to be established through prospective studies.

A novel finding in our study was a relatively high prevalence of pathogenic mutations typically associated with hematologic malignancies, including *KMT2D*, *ASXL1*, and *TET2*, which correlated with inferior survival. This warrants further investigation as it may reflect clonal hematopoiesis of indeterminate potential (CHIP) or previously unrecognized mechanisms contributing to tumor progression in GEP-NENs (31, 32). Functional studies are needed to clarify the impact of these alterations on tumor biology and treatment response. Consistent with the recent study by Balanis *et al.*, which identified a small cell neuroendocrine (SCN)-convergent phenotype across epithelial cancers and hematologic malignancies with shared poor prognosis and drug sensitivities, our findings of hematologic cancer-associated mutations in a subset of GEP-NENs may reflect a similar molecular convergence (33). These mutations could denote an SCN-like transcriptional state, providing a mechanistic basis for their aggressive behavior and guiding new therapeutic strategies beyond traditional tissue-based approaches. The presence of CHIP-associated mutations may have clinical implications for patients receiving PRRT, as preexisting clonal hematopoiesis has been associated with an increased risk of subsequent therapy-related myeloid neoplasms (34).

The enrichment of nuclear and chromatin-associated complexes in both pancreatic and non-pancreatic primaries reveal shared vulnerabilities in genome maintenance and chromatin regulation, with pancreatic primaries favoring epigenetic dysregulation and non-pancreatic exhibiting additional Wnt signaling alterations. These findings underscore the potential for precision therapies targeting mTOR, DNA repair, or Wnt pathways tailored to tumor-specific molecular profiles (35, 36, 37). Currently, belzutifan is the only FDA-approved targeted therapy for NENs, but it applies to less than 1% of patients, highlighting a major unmet need. Future strategies may include earlier or more aggressive treatment approaches in patients with high-risk mutations, such as *KRAS* or *TP53*, and exploration of targeted therapies against *KRAS* or HRD-associated vulnerabilities.

The strengths of our study include a well-annotated clinical cohort with comprehensive genomic profiling and robust statistical analyses adjusting for relevant confounders. This study has several limitations. First, this is a single-center retrospective study with a small sample size, which may limit the statistical power to detect modest associations or assess rare mutations. In addition, because this cohort was derived from patients evaluated in a tertiary multidisciplinary neuroendocrine clinic who underwent clinically indicated NGS, it may be enriched for patients with more advanced or clinically complex disease, potentially limiting the generalizability of our findings. Accordingly, associations between genomic alterations and overall survival, as well as observed co-occurrence and mutual exclusivity patterns, should be interpreted cautiously and viewed as hypothesis-generating rather than definitive prognostic or biological markers. Furthermore, given the small number of mutation-positive tumors, particularly within the KRAS, hematologic malignancy-associated, and ATRX/DAXX groups, we could not fully distinguish the independent contributions of individual co-occurring alterations to the observed survival associations. Second, a key limitation of this study is the use of multiple NGS platforms, each with varying testing characteristics; efforts were made to harmonize classification across platforms using standardized databases, but inter-platform variability may still affect mutation detection and interpretation. Furthermore, NGS was performed as part of routine clinical care at variable time points during the disease course. As a result, we were unable to evaluate the potential effects of prior therapies and clonal evolution on the genomic alterations identified. In addition, the absence of matched normal tissue limits our ability to definitively distinguish tumor-derived somatic mutations from CHIP, particularly for mutations commonly associated with hematologic malignancies. Finally, germline testing was not systematically performed, limiting conclusions about hereditary cancer predisposition syndromes. Therefore, future prospective studies using uniform NGS platforms and matched normal tissue will be required to validate these findings and clarify their clinical significance.

To conclude, our study provides novel insights into the mutational landscape of GEP-NENs, identifying key genomic alterations associated with tumor aggressiveness and survival. The integration of genomic profiling into clinical management could refine risk stratification and guide personalized therapy in this heterogeneous group of tumors. Future studies should focus on the biological mechanisms driving these mutational patterns and evaluate targeted therapies addressing the identified pathways.

## Supplementary materials



## Declaration of interest

The authors declare that there is no conflict of interest that could be perceived as prejudicing the impartiality of the work reported.

## Funding

This research was supported by the McKnight Family Foundation under no specific grant number.

## Author contribution statement

ADJ, PA, and CM conceived the study; designed the methodology; performed data curation, validation, formal analysis, and visualization; and contributed software. ADJ contributed resources, supervised the study, administered the project, and acquired funding. All authors were involved in investigation and wrote, reviewed, and edited the manuscript.

## Data availability

The data underlying this study are not publicly available due to participant privacy and confidentiality considerations. Access to the datasets may be granted to qualified researchers upon reasonable request to the corresponding author; contingent on approval from the appropriate institutional review board; and compliance with all institutional, ethical, and regulatory requirements.
