# Supplementary material for: Comprehensive somatic profiling of gastroenteropancreatic neuroendocrine neoplasms
Source: Endocr Oncol. 2026 Sep 24;6(1):e260044. doi: 10.1530/EO-26-0044 (PMC13641143; doi:10.1530/EO-26-0044)
Supplement: Supplementary file 1 [file EO-26-0044_supplementary_table_1.pdf]

Supplementary Table 1: Distribution of next-generation sequencing (NGS) platforms used in the study cohort

NGS Platform	Number of Patients, n (%)
Johns Hopkins institutional panel	44 (39.6%)
Caris Life Sciences	32 (28.8%)
FoundationOne	24 (21.6%)
Altera	11 (9.9%)
**Total**	**111 (100%)**
